# CdTe-QDs Affect Reproductive Development of Plants through Oxidative Stress

**DOI:** 10.3390/toxics11070585

**Published:** 2023-07-05

**Authors:** Jianhua Wang, Yan Gong, Xiaoyan Yan, Rong Han, Huize Chen

**Affiliations:** 1 Upgrading Office of Modern College of Humanities and Sciences of Shanxi Normal University, Linfen 041000, China; wangjianhuawh@126.com; 2 Shanxi Key Laboratory of Plant Macromolecules Stress Response, Taiyuan 030000, China; yanxy@sxnu.edu.cn; 3 College of Life Science, Shanxi Normal University, Taiyuan 030000, China; crush_bc@163.com

**Keywords:** CdTe-QDs, oxidative stress, abnormal pollen morphology, the defects of pollen vitality, reproductive development

## Abstract

With the continuous development of industry, an increasing number of nanomaterials are widely used. CdTe-QDs is a nanomaterial with good optical properties, but its release into the natural environment may pose a potential threat. The toxicity of nanoparticles in plants is beginning to be questioned, and the effect on phytotoxicity is unclear. In this study, we simulated air pollution and soil pollution (CdTe-QDs concentrations of 0, 0.2, 0.4, 0.8 mmol/L) by spraying and watering the seedlings, respectively. We determined the transport pathways of CdTe-QDs in *Arabidopsis thaliana* and their effects on plant reproductive growth. Spraying CdTe-QDs concentration >0.4 mmol/L significantly inhibited the formation of fruit and decreased the number of seeds. Observation with a laser confocal scanning microscope revealed that CdTe-QDs were mainly transported in plants through the vascular bundle, and spraying increased their accumulation in the anthers and ovaries. The expression level of genes associated with Cd stress was analyzed through RT-qPCR. CdTe-QDs significantly increased the expression levels of 10 oxidative stress-related genes and significantly decreased the expression levels of four cell-proliferation-related genes. Our results reveal for the first time the transport of CdTe-QDs in *Arabidopsis* flowers and demonstrate that QDs can cause abnormal pollen morphology, form defects of pollen vitality, and inhibit pollen tube growth in *Arabidopsis* through oxidative damage. These phenomena ultimately lead to the inability of *Arabidopsis* to complete the normal fertilization process and affect the reproductive growth of the plant.

## 1. Introduction

The rapid development of nanomaterials has brought great benefits to society but also caused many potential risks to the environment [1,2]. With the rapid development of nanotechnology and the application of nanomaterials, nanomaterials inevitably enter the environment [3]. Nanoparticles (NPs) have a size of less than 100 nm and are more likely to enter animals and plants by air, water and soil than other substances of the same type because of their special physical properties. The effects of biotoxicity of nanoparticles on organelles or proteins in cells remain unknown [4,5]. Scholars have focused on the potential harm of nanoparticles to human health and the natural environment [6]. The influence of the environment on plants directly affects the whole ecosystem. Nanoparticles can be absorbed through the plant’s natural nano- or micron-scale openings and accumulate in large quantities in tissues, which not only affect the plant’s own physiological and bio-chemical reactions but also may lead to contamination of the entire food chain [7,8]. We need to elucidate the potential risks of nanoparticles so we can better deal with the environmental problems they caused in the future.

CdTe quantum dots (CdTe-QDs) are semiconductor nanoparticles that are commonly used in light-emitting diodes, lighting, and bioimaging because of their good luminescence properties [9,10,11]. Large-scale industrial applications inevitably lead to the release of CdTe-QDs into the environment, and their environmental impact is of huge concern due to the presence of toxic heavy metal element Cd [12]. Some scholars have found that CdTe-QDs significantly affect the laboratory-scale aquatic microbial food chain, with a 1.4-fold increase in Cd accumulation in *Escherichia coli* and a large accumulation of Cd elements in its predators [13]. In algae, CdTe-QDs produce toxicity in two main ways, namely, by releasing Cd^2+^ or by entering the cell directly. In this regard, CdTe-QDs are more toxic than Cd^2+^. Given the diversity of plants and the environment, the influence of nanoparticles on them also varies. Different varieties of rice have varied abilities to cope with Cd QDs, including Cd accumulation, growth inhibition, antioxidant enzyme activity, and gene level [14]. The biotoxicity of CdTe-QDs is also associated with NP concentration and particle size, and experiments have shown that the toxicity of QDs increases with concentration, while smaller QDs exhibit stronger cytotoxicity [15].

The biotoxicity of Cd-QDs is mainly caused by NPs or released Cd ions, which induce the production of reactive oxygen species (ROS) in cells or tissues and lead to DNA damage. In recent years, many studies have been conducted on the biotoxicity of Cd-QDs based on animal and plant materials. The potential dangers of Cd-QDs to the environment have been analyzed and discussed. CdSe/ZnS QDs in L02 cell lines can induce mitochondrial ROS production and ion loss, activate NLRR3, and eventually lead to the inflammation and dysfunction of liver cells [16]. CdTe-QDs can cause oxidative damage to aquatic shellfish, DNA fracture, and damage to the immune system [17]. The cytotoxicity of CdTe-QDs is enhanced with the increasing Cd ion release rate. UV-B radiation accelerates the release of CdTe-QDs, resulting in the accumulation of oxidative damage in wheat and programmed cell death [18,19]. The transport mechanism and distribution characteristics of QDs in plants have been detected using fluorescence. CdSe/ZnS QDs mainly accumulate in the root and affect the reductive glutathione level, resulting in oxidative stress response [20]. CdTe-QDs can be absorbed by the roots of rice and transferred to the blades by water transport [14]. Plant leaves can absorb elemental Cd from air pollutants, significantly reduce the plant biomass and chlorophyll content by increasing the oxidative stress, and induce a plant stress response by altering the activity of heme oxygenase enzyme [21,22]. NPs also have different absorption modes in different plants that grow in different environments [23].

NPs affect the reproduction and nutrient transmission of plants and animals [24]. The accumulation of TiO_2_-NPs in the human placenta can lead to dysfunction in nutrient transmission, resulting in damages to fetal nerve and reproductive system by inducing oxidative stress, DNA damage, and activation of MAPK signaling pathways [25]. CdSe/ZnS QDs can be reunited on the surface of fish embryos and cause damage to their fluffy membranes, thereby affecting their embryonic development [26]. Defects in the reproductive capacity of *Caenorhabditis elegans* exposed to CdTe-QDs, including dysplasia in proliferation and differentiation, lead to imbalance in egg cells by reducing their number in pachytene and diakinesis [27]. NPs in animals can affect reproductive development by inducing oxidative stress, but few works have reported on the effects of NPs on plant reproductive growth. The mechanisms by which NPs affect reproductive growth in plants are still unknown.

Here, we show that spraying with CdTe-QDs can cause reproductive defects in plants. However, no similar occurrence was observed with the same concentration of CdCl_2_ sprayed. The molecular mechanisms by which QDs lead to reproductive defects in plants are an important objective of this study. Our results provide preliminary insights into the effects of QDs on plant reproductive development through the study of QDs uptake and transport patterns, antioxidant enzyme systems, and regulation of gene expression. These results demonstrate that QDs are more readily absorbed by plants and that QDs can be transported through the vascular bundle to the floral organs of plants, where they affect the antioxidant enzyme system by means of oxidative stress, while altering the expression levels of related genes. Affected by QDs, plants are unable to produce gametes properly and complete the normal fertilization process, ultimately causing defects in embryo development. The results provide evidence for the transportation pathways and potential environmental risks of QDs in plant reproductive organs. 

## 2. Materials and Methods

### 2.1. Characteristics of CdTe-QDs

The CdTe-QDs used in this study were synthesized by the Shanxi key laboratory of Plant Macromolecules Stress Response, Shanxi Normal University, China, and the physicochemical properties of these QDs were determined prior to all experiments [19]. CdCl_2_ was used to prepare Cd^2+^ solutions. The particle size and morphology of CdTe-QDs were characterized using an transmission electron microscope (JEM-2100, JEOL, Tokyo, Japan). The concentration of CdTe-QDs in aqueous solution was set as 0.2, 0.4 and 0.8 mmol/L, and the fluorescence intensity of QDs was observed under the irradiation of ultraviolet lamp with wavelength of 365 nm. Emission spectra of QDs at different concentrations were detected using Agilent Cary Eclipse (Agilent Technologies, Beijing, China) with an excitation light of 365 nm.

### 2.2. Plant Growth and CdTe-QD Treatment

*Arabidopsis thaliana* Columbia-0 ecotype (Col-0) was used for treatment. *Arabidopsis* seeds were sterilized with 1.5% sodium hypochlorite and treated in the dark at 4 °C for 3 days. The seeds were subsequently germinated on 1/2 MS (Murashige and Skoog; Duchefa Biochemie, Haarlem, Holland) agar medium (Phytagel; Sigma, City of Saint Louis, USA) under sterile conditions and grown at 22 °C (day)/18 °C (night) in 50% relative humidity within a photoperiod of 16 h light/8 h dark. The seeds were transferred to a pot after 10 days, and the pots were filled with a mixture of perlite and vermiculite (ratio of 1:3) and were watered with Hoagland nutrient solution until the flowering stage of *Arabidopsis* to start subsequent experiments.

CdTe-QDs with concentrations of control, 0.2, 0.4, and 0.8 mmol/L were used, and 0.8 mmol/L CdCl_2_ was used for Cd ion control treatment. The treatment methods included spraying and watering to simulate, atmospheric and soil pollution, respectively. After *Arabidopsis* had grown to the flowering stage, different concentrations of CdTe-QDs (concentrations of control, 0.2, 0.4 and 0.8 mmol/L) were treated by spraying and watering, repeated after one day interval and phenotypes were observed after 7 days. Before each experiment, we checked the stability of the quantum dots with equipment such as a UV spectrophotometer. We used 0.8 mmol/L concentration of CdCl_2_ as a control. After 7 days, the abnormal flowers of *Arabidopsis* were observed and counted. The pistil was stained with aniline blue, and the anther with stained with Alexander. Silique length and seed number were statistically analyzed after waiting for the silique to mature. Seeds in silique were observed through decolorization treatment with 70% ethanol under Olympus BX53 fluorescence microscope.

### 2.3. ROS Analysis

*Arabidopsis thaliana* was sprayed with different concentrations of CdTe-QDs (concentrations of control, 0.2, 0.4 and 0.8 mmol/L) after growth to flowering stage and repeated after a 1 day interval, and ROS analysis was performed after 1 day. Malondialdehyde (MDA) content was determined using TBA color development. Superoxide dismutase (SOD) activity was measured with the NBT reduction method [28]. Peroxidase (POD) activity was determined based on change in the absorbance of guaiacol at 470 nm. Catalase (CAT) activity was measured using hydrogen peroxide reduction method [29]. 

The flowers were incubated in NBT staining buffer (2 mM NBT and 10 mM NaN_3_ in phosphate buffer, pH 7.0) and incubated in a vacuum for 15 min in a dark environment for the detection of superoxide. Dark processing was conducted for 1 h. Decolorization with 95% alcohol was conducted at 80 °C for 30 min. The samples were photographed using an Olympus BX53 fluorescence microscope.

### 2.4. Pollen Tubes Aniline Blue Staining and Pollen Alexander Staining

The *Arabidopsis thaliana* was treated with CdTe-QDs (0.8 mmol/L) and the effect of quantum dots on pollen tube growth was observed under fluorescence microscopy using aniline blue staining after 1 day. After 5 days of CdTe-QDs (0.8 mmol/L) treatment, the development of gametes in anthers and ovaries was observed under a fluorescent microscope after aniline blue and alexander staining. Pollen tubes were stained with aniline blue. Twenty flowers of stage 12 to 13 in control and treatment groups were selected and fixed with FAA (100 mL of FAA fixative, 5 mL of 38% formaldehyde, 5 mL of glacial acetic acid, and 90 mL of 70% ethanol) at 4 °C overnight. The sample was obtained and subjected to 50%, 30% and 15% and distilled water to cover gradually for 15 to 30 min each time. The sample was softened in 1 M NaOH in a water bath at 50 °C for 4 h and gently rinsed with 108 mM potassium phosphate solution three times. The sample was added with 0.1% (*w*/*v*) water-soluble aniline blue solution (0.5 g of water-soluble aniline blue solution was added to 108 mM potassium phosphate solution up to a constant volume of 500 mL) and incubated in a dark environment for 10–15 min. The growth of pollen tubes was observed with an Olympus BX53 fluorescence microscope under 10× objective, excitation wavelength of 420–490 nm, and emission wavelength of 510 nm [30].

Anthers and pollen were Alexandria stained. Twenty fresh flowers from control and CdTe-QDs treatment groups were selected, and petals and pistils were carefully removed. About two drops of Alexander Dye (Solarbio) were added to the anther on the slide and thoroughly mixed. The slide was covered with a coverslip and stained for 1–5 h. Excess stain was absorbed, and the specimen was observed under an Olympus BX53 fluorescence microscope.

### 2.5. Microscopy

The *Arabidopisis thaliana* was treated by spraying CdTe-QDs (0.8 mmol/L), and the transport and distribution of QDs in the *Arabidopisis thaliana* organs was observed using laser confocal 2 days later. The samples were observed using a confocal (Olympus FV1000) microscope with 1.4 NA and 60× oil immersion objective. CdTe-QDs were visualized using 488 nm laser excitation and 500 to 550 nm spectral detection. Images were collected at a size of 512 × 512 pixels by using the FV10-ASW 4.2 viewer software. Pollen tubes stained with aniline blue and pollens stained with Alexander dye were observed with fluorescence microscopy (BX53, Olympus, Tokyo, Japan). Images were acquired using an Olympus DP71 CCD camera. Flowers, siliques and seeds were observed with a Zeiss Axio Zoom V16 Materials Stereo Zoom Microscope, and images were acquired using Zeiss Axiocam 506 color camera. Seeds within the silique were observed using an Olympus BX53 fluorescence microscope, and images were acquired using an Olympus DP71 CCD camera. Adobe Photoshop CS5 was used to splice the collected images.

### 2.6. Expression Analysis

The *Arabidopsis thaliana* was sprayed with different concentrations of CdTe-QDs (concentrations of control, 0.4 and 0.8 mmol/L) after growth to flowering stage and repeated after a 1 day interval. Expression analysis was performed after 1 day. RNA was prepared from the flower for RT-qPCR. Total RNA was extracted using a TransZol Up Plus RNA kit (TransGen Biotech, Beijing, China). Total RNA was reverse-transcribed into cDNA by using *TransScript*^®^ One-Step gDNA Removal and cDNA Synthesis SuperMix kit (TransGen Biotech). RT-qPCR was performed on a QuantStudio 3 Real-Time PCR System (Applied Biosystems) with *PerfectStart*^TM^ Green qPCR SuperMix (TransGen Biotech). The expression of each gene was standardized using the expression level of *ACTIN7*. Data were derived from three experimental replicates, using approximately 15 plants for each experiment. The primer sequences used are listed in Supplementary Table S1.

The Ct value of each treatment group was obtained through RT-qPCR, and the relative expression level of each treatment group was calculated using the 2^—ΔΔCt^ method [31]. The calculation formula is as follows:

2^—ΔCt Control^ = 2^—(Ct Control A−Ct Control B)^

2^—ΔCt Sample^ = 2^—(Ct Sample A−Ct Sample B)^

2^—ΔΔCt^ = 2^—ΔCt Control^/2^—ΔCt Sample^

### 2.7. Statistical Analysis

All statistical analyses were conducted on data obtained from three or more biologically independent experiments (as specified in the Figure legends), and all data were expressed as means ± standard deviations (SD). Analyses were performed with SPSS software, using a one-way ANOVA followed by Tukey’s test. *p* values < 0.05 were considered statistically significant [32]. 

## 3. Results

### 3.1. Physicochemical Properties of CdTe-QDs

**Figure 1:**
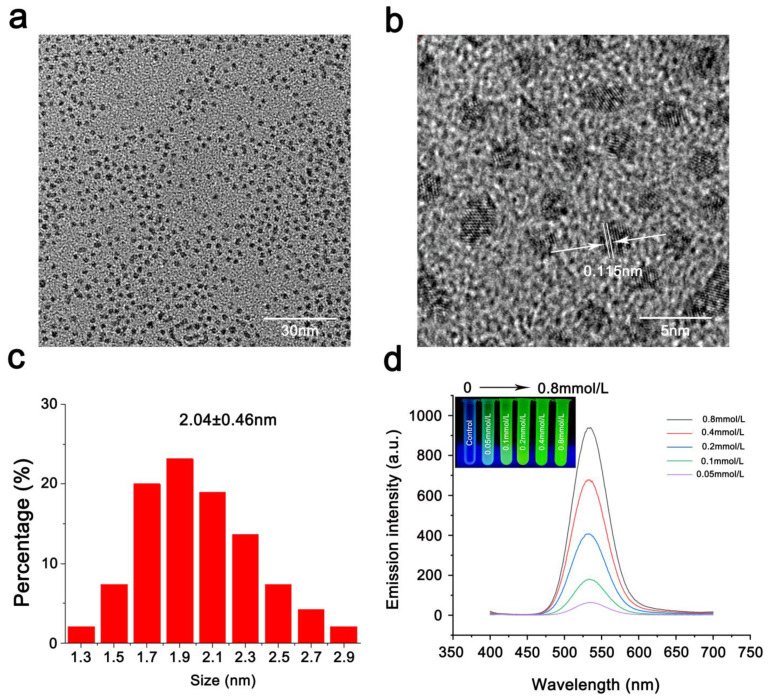
Characterization of CdTe-QDs. (**a**,**b**) TEM image of synthesized CdTe-QDs. (**c**) CdTe-QDs have an average diameter of 2.04 ± 0.46 nm. (**d**) Fluorescence intensity of CdTe-QDs at different concentrations. Emission fluorescence spectra of CdTe-QDs at different concentrations excited by 365 nm. In the upper left corner are fluorescence images of CdTe-QDs with different concentrations under a UV lamp (365 nm).

CdTe-QDs have an average diameter of 2.04 ± 0.46 nm (Figure 1a,c), good water solubility, a narrow fluorescence emission spectrum and a maximum emission wavelength of 531 nm (Figure 1d). The QD solution emitted green fluorescence under UV lamp irradiation at 365 nm excitation. We tested the fluorescence intensity of different concentrations of QDs under UV lamps and found that the fluorescence became stronger with increasing concentration (Figure 1d). A fluorescence spectrophotometer was used to detect the emission spectra of different concentrations of QDs under a 365 nm excitation light. The maximum emission intensity of QDs of different concentrations was approximately 531 nm; the intensity of emission increased with increasing concentration of QDs (Figure 1d). Samples (0.2, 0.4 and 0.8 mmol/L) with high fluorescence intensity were selected as treatment concentrations in subsequent experiments.

### 3.2. Effect of Different Modes of CdTe-QDs Treatment on Arabidopsis thaliana Growth

The effects of CdTe-QDs on the reproductive growth of *A. thaliana* were analyzed using spraying and watering to simulate atmospheric pollution and soil contamination, respectively. *Arabidopsis* at the flowering stage was treated with different concentrations of QDs (control, 0.2, 0.4 and 0.8 mmol/L). The types and quantities of various anomalies of flowers treated by the two methods were determined. 

**Figure 2:**
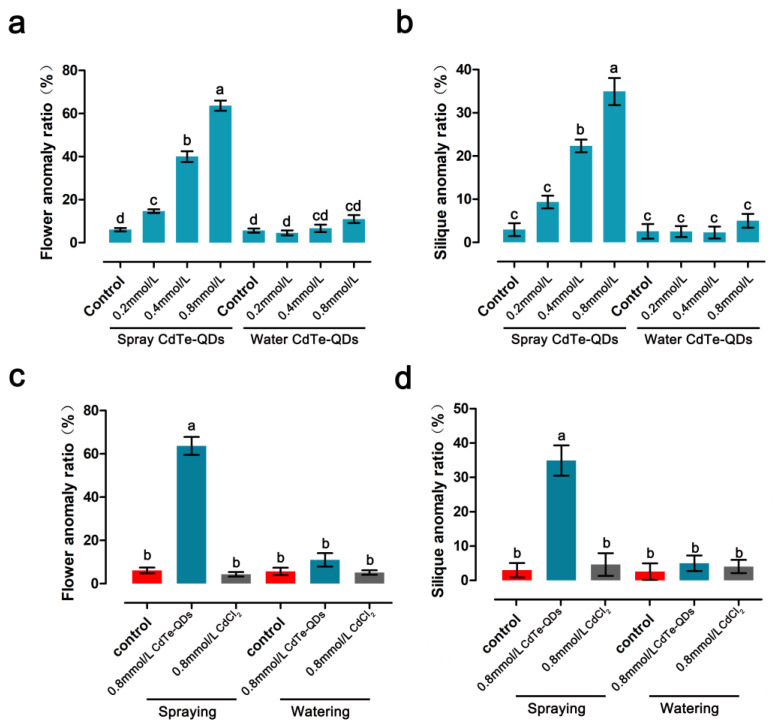
Different treatments of CdTe-QDs lead to reproductive growth abnormalities in *Arabidopsis thaliana*. (**a**) Proportion of *Arabidopsis* flower abnormalities caused by two treatments with different concentrations of CdTe-QDs. (**b**) Proportion of *Arabidopsis* silique abnormalities resulting from two treatments with different concentrations of CdTe-QDs. (**c**,**d**) Compared with Cd^2+^, CdTe-QDs can significantly affect the growth and development of *Arabidopsis* flowers. The letters a to d indicate statistically significant differences determined by Tukey’s test (*p* < 0.05). The error bars represent SD (*n* = 15).

Spraying of QDs significantly affected the growth and development of *Arabidopsis* flowers at all stages of development and significantly changed the petal morphology. The proportion of petal abnormalities increased with increasing concentration of QDs compared to the control; the values were 14.67% for 0.2 mmol/L QDs treatment, 40% for 0.4 mmol/L QDs treatment, and 63.67% for 0.8 mmol/L QDs treatment (Figure 2a). Compared with the spray treatment, watering QDs did not significantly affect flower development, and the proportion of abnormal petals was not significantly different from the control (Figure 2a). This finding could be related to the mode of transport of QDs, which will need to be further investigated. The proportions of abnormal siliques were determined. Spraying of QDs caused abnormal silique morphology in certain proportions: about 9.37% for 0.2 mmol/L, 22.33% for 0.4 mmol/L, and 34.93% for 0.8 mmol/L. No obvious abnormality was detected in the samples subjected to watering of QDs (Figure 2b). The effect of 0.8 mmol/L CdCl_2_, as control, on the flowering growth and development of *A. thaliana* was evaluated. CdCl_2_ had no significant effect on the reproductive growth of *A. thaliana* (Figure 2c,d).

Based on the above results, spraying CdTe-QDs significantly affected the reproductive growth process of *Arabidopsis thaliana*, and the follow-up experiments were mainly carried out in this way. According to statistical analysis, 0.4 mmol/L and 0.8 mmol/L significantly affected the reproductive growth of *Arabidopsis thaliana*, and these two concentrations were mainly used in subsequent experiments.

### 3.3. Distribution of CdTe-QDs in Plant Tissues

CdTe-QDs used in this study emit green spontaneous fluorescence through ultraviolet light, a physical feature that makes it easier for us to observe how QDs travel and are distributed in plants. The *Arabidopisis thaliana* was treated by spraying CdTe-QDs, and the transport and distribution of QDs in the *Arabidopisis thaliana* organs was observed with laser confocal 2 days later. 

**Figure 3:**
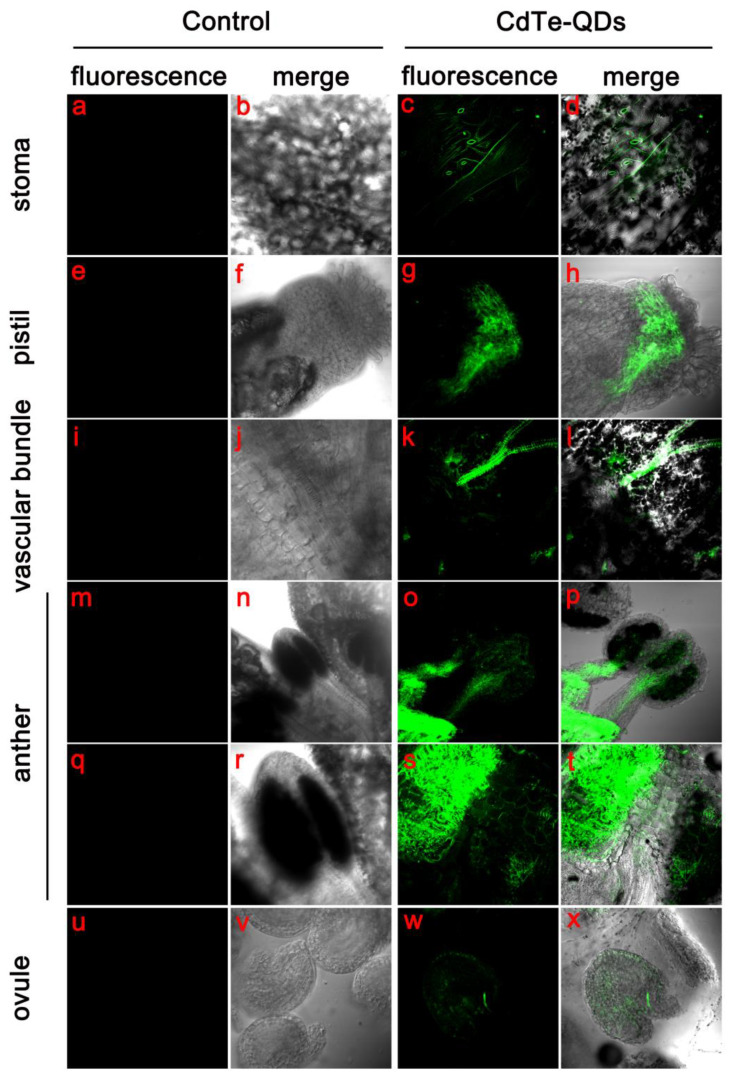
Distribution of CdTe-QDs in *Arabidopsis* flower using laser confocal microscopy. The first and third columns are fluorescence images of 488 nm excited CdTe-QDs, and the second and fourth columns are superimposed images of bright-field and fluorescence channels. (**a**–**d**) Accumulation of QDs can be observed in stomata. (**e**–**h**) Accumulation of QDs can be observed at the stigma and translocation to other sites via the vascular bundle of the pistil. (**i**–**l**) Vascular bundles may be one of the pathways by which QDs are transported in floral organs, and we also observed transport of QDs in vascular bundles at other sites. (**m**–**t**) Accumulation of QDs was observed in anthers, and large amounts of QDs were observed on the surface of pollen sacs and in vascular bundles in filaments. (**u**–**x**) QDs were transported into ovules by vascular transport. Bars = 50 μm.

By looking at multiple locations in the flower organ, we detected a fluorescent signal from CdTe-QDs. In the epidermis of the calyx and petals, we observed a large number of QD aggregations in the stomata (Figure 3c). QDs were also observed in the stigmas of the pistil and metastasized to other sites through the vascular bundles in the ovary (Figure 3g,k). We hypothesized the main transport mode of CdTe-QDs in floral organs may be mainly dependent on vascular bundles. To confirm this, we observed the localization of QDs in *Arabidopsis* anthers and ovules. The results showed that QDs could be transported to the flower medicine and embryo beads of *Arabidopsis* by the vascular bundle (Figure 3p,x). QDs could be observed in the vascular bundle in the center of the flower wire (Figure 3o), the distribution of QDs could be observed on the surface of the pollen sac (Figure 3s). At the same time, it was observed that QDs in ovules can also be transported in a vascular-bundle-dependent manner (Figure 3w). In addition, the transport of watering CdTe-QDs in *Arabidopsis* was observed, and it was found that QDs could be absorbed by roots, but they were not transported to the above-ground part through vascular bundles or other approaches (Figure S1). 

The results of our observation revealed for the first time the transport pattern and distribution characteristics of CdTe-QDs in the *Arabidopsis* flower. The primary mode of uptake of QDs in the floral organ is through the stigma, skin and pores into plant tissues and transport to the anther, ovary and ovule via vascular bundles.

### 3.4. Effects of CdTe-QDs on Plant Reproductive Growth

Observations with a laser confocal microscope showed that spraying CdTe-QDs can cause QDs to accumulate in the floral organs of *Arabidopsis thaliana*. The results prove that QDs accumulate in both male and female gametes of flowers. Due to the potential biological toxicity of CdTe-QDs, we analyzed the effects of spraying QDs on the reproductive growth of *Arabidopsis*.

**Figure 4:**
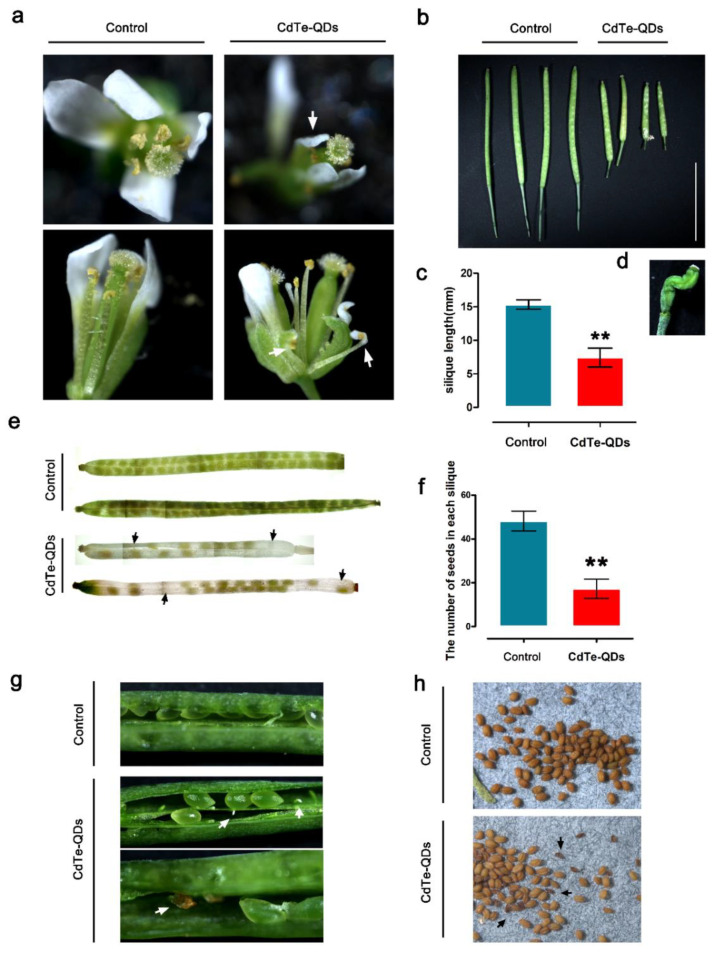
Effect of CdTe-QDs on *Arabidopsis* reproductive growth. (**a**) We observed the changes in flower morphology at 13 stages after QDs treatment. Compared with the control, the calyx and petals shrank, and the filaments appeared twisted. The white arrow indicates an abnormal phenotype. Bar = 1 mm. (**b**,**c**) The length of the silique changed after QDs treatment. Through the analysis of significant difference, QDs obviously affected the growth of silique. “**” indicates statistically significant differences (*p* < 0.01). Bar = 10 mm. The error bars represent SD (*n* = 15). (**d**) Some siliques shrunk after QDs treatment. (**e**) CdTe-QDs have a significant effect on the seed setting rate of *Arabidopsis*. Bar = 10 mm. (**f**) The number of seeds in each silique was counted. Through the analysis of significant differences, QDs significantly reduced the number of seeds. “**” indicates statistically significant differences (*p* < 0.01). The error bars represent SD (*n* = 15). (**g**) The seed vacancies in the siliques, mainly caused by unfertilized ovules and aborted seeds. The white arrows indicate abnormally developed seeds. Bar = 500 μm. (**h**) QDs treatment caused many shriveled seeds (black arrows). Bar = 500 μm.

After CdTe-QDs treatment, the *Arabidopsis* flower morphology showed obvious distortion; the petals shrank to different degrees, the filaments of the stamens were twisted, the pollen was less full than the control, and the amount of pollen attached to the stigma of the pistil has also been reduced (Figure 4a). In addition, through the statistics of silique length, it is found that QDs treatment significantly affects the length of *Arabidopsis* silique (Figure 4b,c). Some of the siliques are deformed (Figure 4d). The silique was transparentized, and the growth and development of the seeds in the silique were observed. It was observed that QDs significantly reduced the number of seeds in the silique. In the flowers of the treatment group, about 17 mature seeds could be observed in each silique, a large number of seed vacancies could be seen in each silique compared with the control, and approximately 48 mature seeds could be observed for each silique in the control group, with fewer seed vacancies (Figure 4e,f). Peeling off the siliques treated with CdTe-QDs, we found that there were two main reasons for the vacancies: the ovules that failed to be fertilized and the aborted seeds (Figure 4g). The condition of the seeds after CdTe-QDs treatment was observed, and by comparison, the QDs caused a large number of shriveled and wrinkled seeds (Figure 4h). 

Based on the above results, CdTe-QDs has a significant inhibitory effect on the reproductive growth of *Arabidopsis.* QDs may affect the growth and development of male and female gametes through the transfer of QDs in the floral organs, and ultimately lead to a reduction in *Arabidopsis* seed quality. The reduction in seed quality has obvious biological toxicity to the reproductive growth of plants.

**Figure 5:**
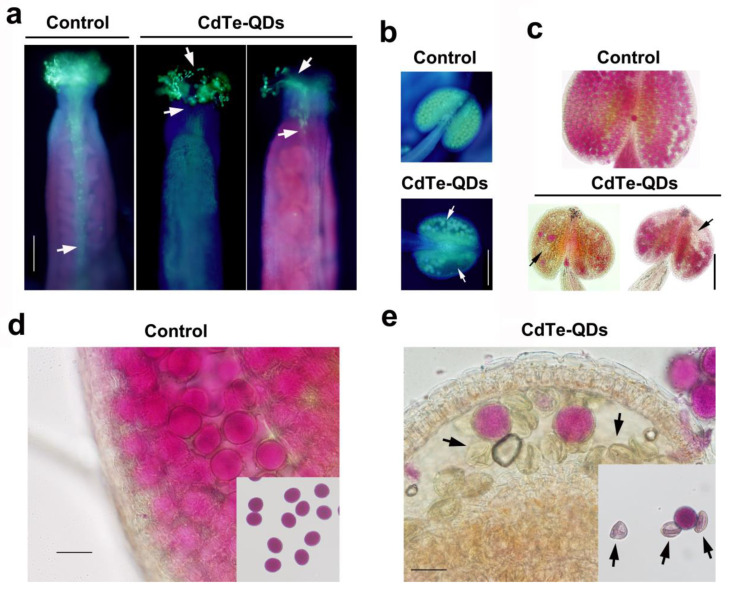
Observation of aniline blue and Alexander staining. (**a**,**b**) Aniline blue staining observed the growth of pollen tubes in the pistil after fertilization. It can be seen that CdTe-QDs affected the growth of pollen tubes to the ovary. Compared with the control, the quantity of pollen in the anthers treated with QDs was also significantly reduced. Bars = 200 μm. The white arrow in Figure a indicates the growth of the pollen tube. The white arrow in Figure b indicates an abnormally developed pollen sac (**c**) The anthers dyed with Alexander dye solution. It can be found that QDs caused the abnormal development of pollen and the quantity of pollen decreased. Bar = 200 μm. (**d**,**e**) The vigor and morphology of pollen changed after QDs treatment (Black arrow). Bars = 50 μm.

In order to more clearly reveal the reproductive toxicity of CdTe-QDs to plants, the growth of pollen tubes in the ovary of *Arabidopsis thaliana* after pollination was observed with aniline blue staining under a fluorescence microscope. After pollination, it was observed that the pollen tube of the normal treatment group has grown through the pistil to each ovary. In contrast, the amount of pollen on the stigma of the pistil treated with CdTe-QDs is lower, and the growth of the pollen tube is obviously affected. Only a small number of pollen tubes grew through the pistil to the ovary, which may be related to the biological toxicity of QDs in the pistil (Figure 5a). Normal pollen tubes can reach the ovule to complete fertilization, but QDs treatment may cause abnormalities in this process (Figure S2). Reduced pollen counts were also found in the mature uncracked anthers of the QDs treatment group (Figure 5b). Alexander staining was carried out on the flowers of stages 12~13. The staining results confirmed that CdTe-QDs treatment would indeed lead to a decrease in pollen number and pollen activity (Figure 5c). It can be observed in the pollen sacs in the QDs treatment group that the morphology of pollen was also significantly affected (Figure 5d,e). These results further confirmed the reproductive toxicity of CdTe-QDs in plants.

### 3.5. Oxidative Stress Marker Assay

**Figure 6:**
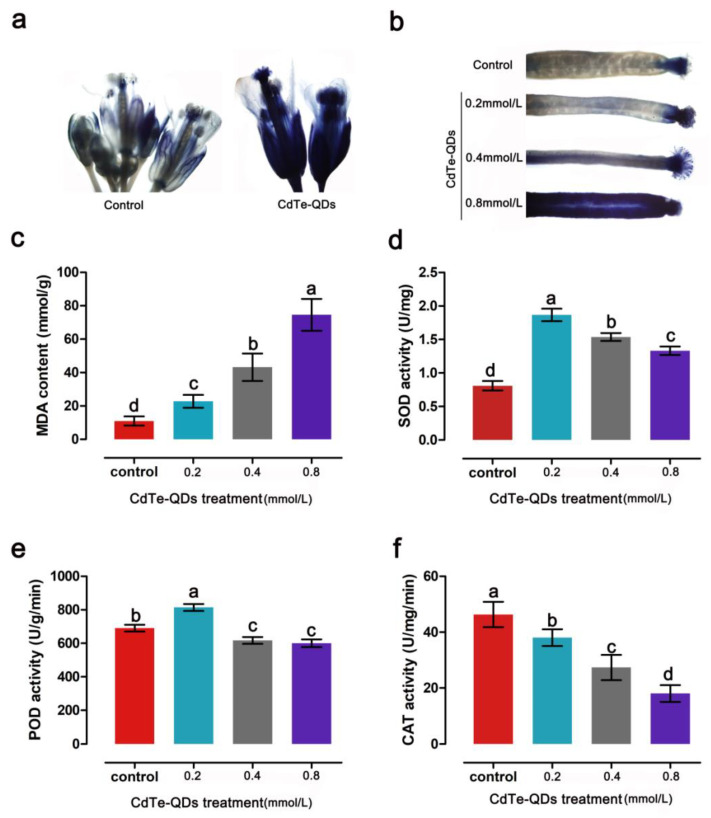
The response of *Arabidopsis* flowers to oxidative stress. (**a**) The results of NBT staining showed that CdTe-QDs treatment caused a large amount of superoxide to accumulate in the flowers. (**b**) As the concentration increased, the accumulation of superoxide increased. (**c**) The content of MDA increased significantly with increasing concentration. (**d**,**e**) Low concentration of CdTe-QDs improved the activity of SOD and POD, and high concentrations of QDs inhibited the activity of SOD and POD. (**f**) CdTe-QDs inhibited the activity of CAT. Error bars represent SDs and different letters indicate significant differences at *p* < 0.05. The error bars represent SD (*n* = 15).

Cadmium affects plants mainly through oxidative stress. We speculate that CdTe-QDs produce superoxide in the floral organs of *Arabidopsis thaliana*, which in turn triggers oxidative stress and leads to abnormal plant reproductive development. To confirm our hypothesis, NBT staining was performed on the floral organs of *Arabidopsis* to detect superoxide. In the floral organs of the control group, a small amount of NBT was observed on the calyx and stigma, while after CdTe-QDs treatment, a large amount of superoxide was detected in the entire floral organ (Figure 6a). At the same time, it was found that as the concentration of CdTe-QDs increases, more superoxide accumulates in the siliquas of *Arabidopsis* (Figure 6b).

The influence of CdTe-QDs on the antioxidant system was analyzed, and oxidative stress markers such as MDA, SOD, POD and CAT were determined. The results showed that, as the concentration of QDs increased, the MDA in floral organs gradually increased, indicating that membrane peroxidation damage gradually increased (Figure 6c). The results of SOD and POD activity show that low concentrations of CdTe-QDs can activate the antioxidant system and help plants cope with the damage caused by oxidative stress, but as the concentration of QDs increases, the activity of antioxidant enzymes is significantly inhibited (Figure 6d,e). The results of CAT activity showed that CAT activity was significantly inhibited as the CdTe-QDs concentration increased (Figure 6f).

### 3.6. CdTe-QDs Changed the Oxidative Damage and Growth and Development Gene Expression Patterns in Arabidopsis Floral Tissue

**Figure 7:**
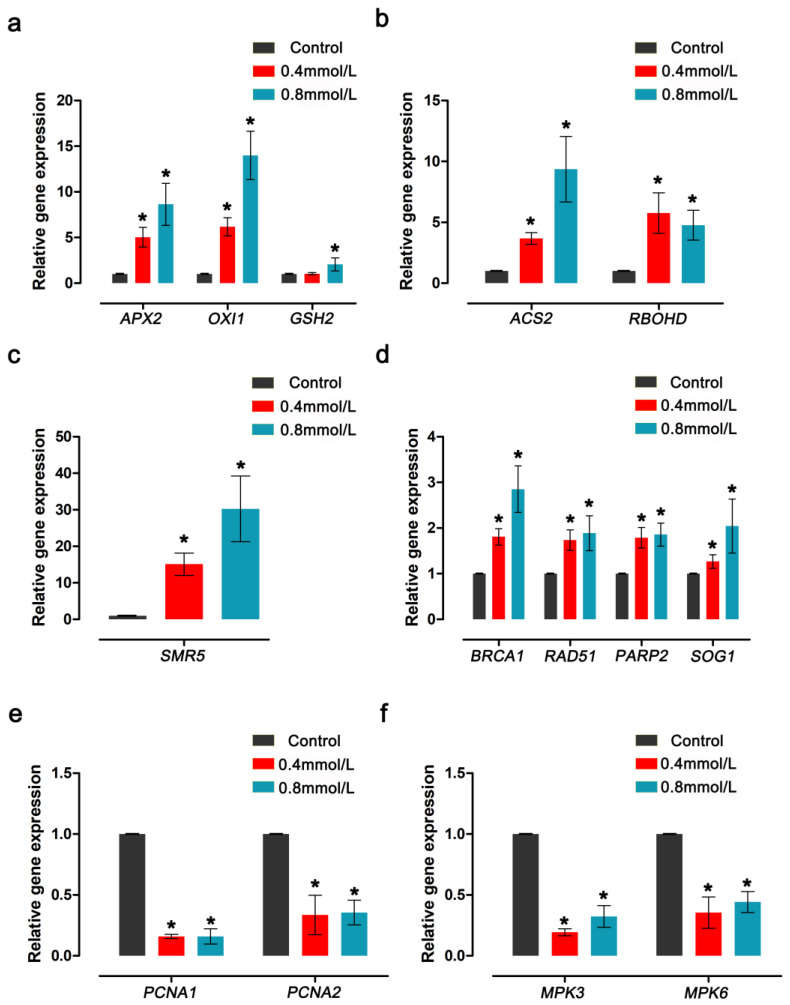
The effect of CdTe-QDs on *Arabidopsis* floral genes was detected using RT-qPCR. (**a**,**b**) The expression levels of the oxidative-stress-related genes *APX2*, *OXI1*, *GSH2*, *ACS2* and *RBOHD* were significantly up-regulated with the increase in QDs concentration. (**c**,**d**) The expression levels of DNA-damage-repair-related genes *SMR5*, *BRCA1, RAD51*, *PRAP2* and *SOG1* were significantly up-regulated as the concentration of QDs increased. (**e**,**f**) The expression levels of *PCNA1*, *PCNA2*, *MPK3* and *MPK6* related to cell proliferation and embryonic development were significantly down-regulated with the increase in QDs concentration. The * indicates statistically significant differences according to Tukey’s test (*p* < 0.05), and the internal reference gene is *AtACTIN7.* Error bars represent the SD of three biological replicates (*n* = 3).

The expression patterns of antioxidant enzymes, DNA-damage-response- and growth-related genes in *Arabidopsis thaliana* floral tissues treated by CdTe-QDs were detected using RT-qPCR, providing evidence for the effect of CdTe-QDs on plant growth and development. The results showed that CdTe-QDs significantly up-regulated the expression levels of 10 of these genes, and the expression levels of these genes increased with increasing concentrations of QDs. The other four genes were significantly down-regulated after CdTe-QDs treatment (Figure 7).

The analysis revealed that the 10 genes up-regulated by CdTe-QDs treatment were mainly genes related to the oxidative damage response and DNA damage response. As shown in Figure 6a, the expression levels of ASCORBATE PEROXIDASE 2 (*APX2*) and OXIDATIVE SIGNAL-INDUCIBLE 1 (*OXI1*) after treatment with 0.4 mmol/L CdTe-QDs increased by about 5~7 times compared with the control. After treatment with 0.8 mmol/L CdTe-QDs, the gene expression level was increased even more compared with the control; *APX2* increased by about 9-fold and *OXI1* increased by about 13-fold (Figure 7a). The gene expression of GLUTATHIONE SYNTHETASE 2 (*GSH2*) did not change significantly under the treatment of 0.4 mmol/L QDs. With the increase in QDs concentration, the gene expression level was significantly up-regulated by about two-fold (Figure 7a). 1-AMINO-CYCLOPROPANE-1-CARBOXYLATE (ACC) SYNTHASE 2 (*ACS2*), a gene related to ethylene synthesis, also changed significantly under CdTe-QDs treatment, and the gene expression level was significantly up-regulated as the concentration increased (Figure 7b). The gene levels of RESPIRATORY BURST OXIDASE HOMOLOGUE D (*RBOHD*) before and after treatment increased by about four to six-fold, and the difference between different concentrations was not obvious. The five genes are mainly involved in response to oxidative damage to the plants, to enhance plant resistance to oxidative damage by upregulation of such genes.

The expression levels of some genes associated with DNA damage are also significantly up-regulated. Among them, the gene of SIAMESE-RELATED 5 (*SMR5*), a member of the plant CDK inhibitor family, has been significantly up-regulated. The gene expression level of 0.4 mmol/L treatment concentration was about 15 times that of the control group. The treatment concentration of 0.8 mmol/L increases the gene expression level of *SMR5* by about 33 times (Figure 7c). It can be seen that, as the concentration of CdTe-QDs increases, the level of DNA damage caused by oxidative stress increases significantly. After CdTe-QDs treatment, BREAST CANCER SUSCEPTIBILITY1 (*BRCA1*), *RAD51*, POLY (ADP-RIBOSE) POLYMERASE 2 (*PARP2*), SUPPRESSOR OF GAMMA RADIATION 1 (*SOG1*) and other genes related to DNA damage repair were also significantly up-regulated (Figure 7d).

The expression level of cell proliferation-related genes was detected using RT-qPCR. It was found that CdTe-QDs significantly reduced the expression levels of four genes related to growth and development. Among them, PROLIFERATING CELLULAR NUCLEAR ANTIGEN 1 and 2 (*PCNA1* and *PCNA2*) significantly down-regulated gene expression levels after treatment with different concentrations (Figure 7e). The expression levels of MITOGEN-ACTIVATED PROTEIN KINASE 3 and 6 (*MPK3* and *MPK6*), which are related to embryonic development, were also significantly down-regulated (Figure 7f). In summary, CdTe-QDs can induce the expression of genes related to oxidative stress and DNA damage, and down-regulate the expression of genes related to growth and development, eventually leading to the inhibition of growth and development by QDs. The changes in gene levels induced by these QDs further prove its toxicity in *Arabidopsis thaliana* in reproductive growth.

## 4. Discussion

With the wide application of nanotechnology, the impact of nanomaterials on the environment deserves more attention, especially the impact on plants [33,34]. CdTe-QDs are often used in biological monitoring because of their excellent luminescence properties, so the potential toxicity of CdTe-QDs to the environment is worthy of our attention [35,36]. This study revealed the reproductive toxicity of CdTe-QDs in *Arabidopsis*. Our results demonstrate that QDs can enter the plant through the epidermis, stomata and stigma and travel to anthers and ovules via vascular bundles, inducing oxidative stress, altering the expression pattern of related genes, and ultimately leading to abnormal plant embryo development.

The pollution of air, water and soil has the greatest impact on plants [37]. We simulated precipitation pollution and soil pollution to analyze the effects of CdTe-QDs on the reproductive growth of *Arabidopsis*. The results showed that the spraying of QDs in the flowering period of *Arabidopsis thaliana* had a more significant impact on reproductive growth. Previous research results indicate that NPs can enter plants through the symplast and apoplast pathways [38]. In *Arabidopsis*, CdSe/ZnS can be absorbed by the root system but not transferred to the leaves [20], our results also show that watering methods have little effect on reproductive growth of *Arabidopsis*, and CdTe-QDs cannot be detected in floral organs. The direct spray mode found that CdTe-QDs accumulate more in floral organs, and CdTe-QDs can have a compound effect with UV-B radiation, which is more toxic to the reproductive growth of plants [19,39].

The expression patterns of oxidative-stress-, DNA-damage-, growth- and development related-genes in floral organs were analyzed. The ROS produced by CdTe-QDs induces oxidative stress and activates the expression of plant antioxidant enzyme-related genes and DNA-damage-repair-related genes. APX2 is a typical active oxygen scavenging enzyme. CdTe-QDs treatment leads to a significant up-regulation of *APX2*, which may be related to the rapid accumulation of ROS. The APX enzyme can use ascorbic acid to reduce hydrogen peroxide to water, thereby reducing the risk of oxidative damage [40,41]. Oxidation signal-inducing factor *OXI1* is an important ROS defense gene, which plays a defense function by triggering the downstream MAPK cascade reaction [42]. GSH2 can help plants deal with nano-toxicity, especially detoxification of oxidative stress caused by heavy metals [43]. In addition, CdTe-QDs also induced high expression of *ACS2* and *RBOHD*. Up-regulation of *ACS2* can lead to changes in petal morphology, and at the same time regulate the synthesis of ethylene to participate in plant stress responses [44]. The *RBOH* gene is involved in plant defense response during abiotic and biotic stress. *RBOHD* is involved in the production of ROS and related signal pathways in plants [45,46,47]. The significant up-regulation of these oxidative-stress-related genes proves that CdTe-QDs mainly affect plant growth in the form of oxidative damage, and the degree of oxidative damage is positively correlated with the concentration of QDs.

The oxidative stress response of plants may lead to DNA damage. Plants can change the cell cycle or undergo programmed cell death through the DNA damage response pathway, which ultimately affects growth and development. From our results, we can see that *SOG1*, *RAD51*, *BRCA1*, *PRAP2* and *SMR5* are significantly up-regulated in DNA-damage-repair-related genes [48,49,50], indicating that CdTe-QDs caused serious damage to plant DNA. The significant down-regulation of genes related to cell proliferation and embryonic development such as *PCNA1*, *PCNA2*, *MPK3* and *MPK6* also indicated that QDs significantly inhibited plant reproductive development [51,52].

## 5. Conclusions

The present investigation explores the effect of CdTe-QDs on *Arabidopsis* reproductive growth. Our results show that spraying CdTe-QDs on the floral organs of *Arabidopsis thaliana* in the form of simulated precipitation pollution can cause plant oxidative stress. Laser confocal scanning microscopy revealed that CdTe-QDs were mainly transported in plants through the vascular bundle, and spraying increased their accumulation in the anthers and ovaries. CdTe-QDs significantly increased the expression levels of 10 oxidative-stress-related genes and significantly decreased the expression levels of four cell-proliferation-related genes, leading to *Arabidopsis* pollen sterility, abnormal pollen tube growth, embryonic abortion, etc., which eventually affects the reproductive growth of plants. The extensive use of CdTe-QDs in various fields has brought us great convenience, but its potential environmental hazards should also be taken into account.

## Data Availability

The authors declare that the data supporting the findings of this study are available within the article and its additional information files.

## Supplementary Materials

The following are available online at https://www.mdpi.com/article/10.3390/toxics11070585/s1, Figure S1: The distribution of QDs can be observed in Arabidopsis thaliana roots by irrigating CdTe-QDs. Figure S2: Effect of CdTe-QDs on the growth of pollen tube of *Arabidopsis thaliana*. The pollen tube of QDs treatment group could not reach the ovule normally to complete fertilization. Supplemental Table S1: List of primers used in this study.
