# Supplementary material for: CdTe-QDs Affect Reproductive Development of Plants through Oxidative Stress
Source: Toxics. 2023 Jul 5;11(7):585. doi: 10.3390/toxics11070585 (PMC10386043; doi:10.3390/toxics11070585)
Supplement: Supplementary file 1 [file toxics-11-00585-s001.zip › toxics-2360361-supplementary.pdf]

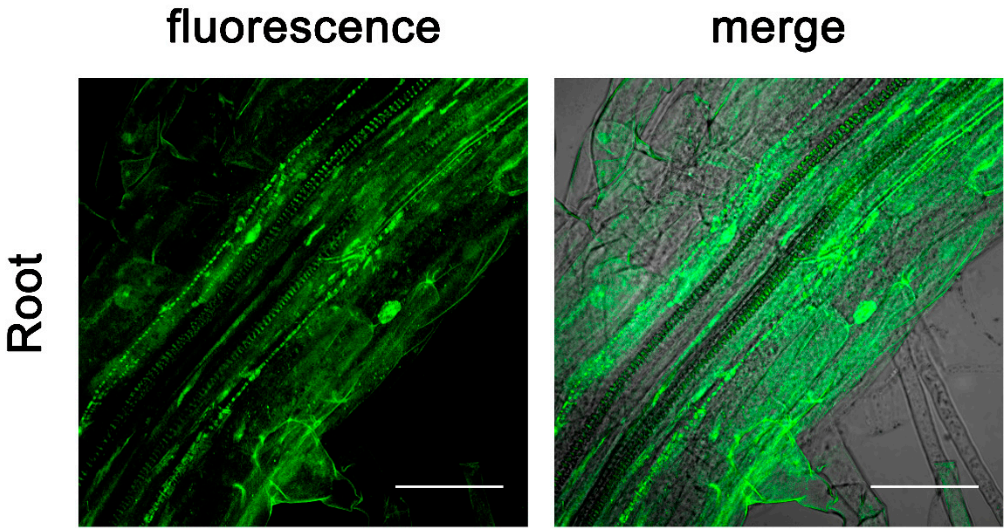

Figure. S1 The distribution of QDs can be observed in *Arabidopsis thaliana* roots by irrigating CdTe-QDs. Bars = 50μm.

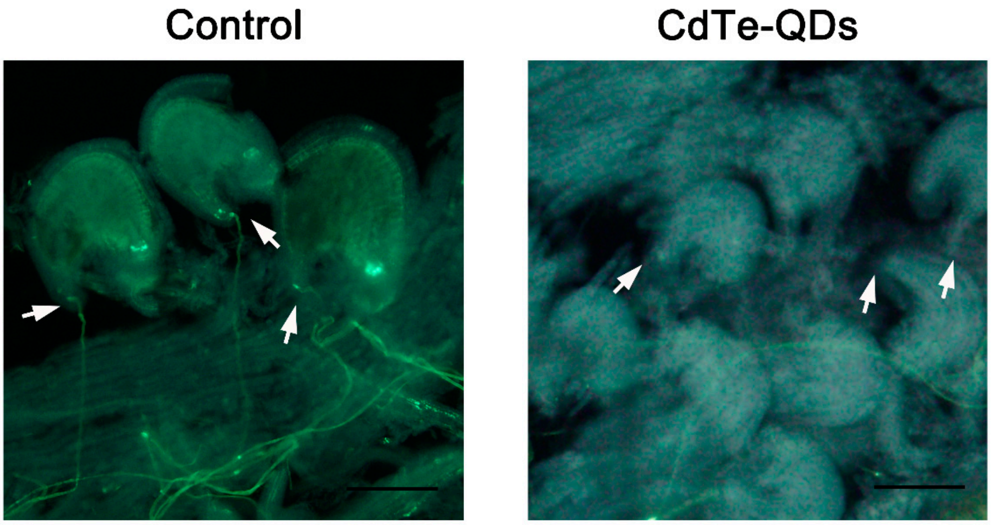

Figure. S2 Effect of CdTe-QDs on the growth of pollen tube of *Arabidopsis thaliana*. The pollen tube of QDs treatment group could not reach ovule normally to complete fertilization. Bars = 200μm.

Supplemental Table S1: list of primers used in this study.

Primer	Sequence
SOG1-F	AGTGGTGTGGAAGAGCAACC
SOG1-R	GCAATCCTGGCCAATCATCAA
SMR5-F	CAGCATATCCGCCTTGTCCA
SMR5-R	CTGCTACCACCGAGAAGAACAAGT
PARP2-F	ATCGGAGGTGATTGATCGGTATG
PARP2-R	AAATCATGAGGTATCACTGTGTAGAACTCT
BRCA1-F	GTGAACCTGTCTCTGCGGAT
BRCA1-R	TCCGGCTTCTTGTCAACTCC
RAD51-F	GTCCAACAACAAGACGATGAAGAA
RAD51-R	AACAGAAGCAATACCTGCTGCC
RBOHD-F	AACTCTCCGCTGATTCCAACG
RBOHD-R	TGGTCAGCGAAGTCTTTAGATTCCT
APX2-F	TTGCTGTTGAGATCACTGGAGGA
APX2-R	TGAGGCAGACGACCTTCAGG
OXI1-F	TAGAGGATCGAACCGGAAAG
OXI1-R	GACCCTTGATTTCCTCAACG
MPK3-F	GACGTTTGACCCCAACAGAA
MPK3-R	TGGCTTTTGACAGATTGGCTC
MPK6-F	TAAGTTCCCGACAGTGCATCC
MPK6-R	GATGGGCCAATGCGTCTAA
ACS2-F	CATGTTCTGCCTTGCGGATC
ACS2-R	ACCTGTCCGCCACCTCAAGT
GSH2-F	GGACTCGTCGTTGGTGACAA
GSH2-R	TCTGGGAATGCAGTTGGTAGC
PCNA1-F	GCAAAGATCTCAGTAGCATTGG
PCNA1-R	CGATAAGCTGATTGTCACTGTG
PCNA2-F	GAAGTTTTCAACAGCAGGTGAT
PCNA2-R	CTTCTTCTTCAATCTTAGGCGC
Actin7-F	AGGCACCTCTTAACCCTAAAGC
Actin7-R	GGACAACGGAATCTCTCAGC
